# Supplementary material for: Exploring the Effects of Metabolism-Disrupting Chemicals on Pancreatic α-Cell Viability, Gene Expression and Function: A Screening Testing Approach
Source: Int J Mol Sci. 2023 Jan 5;24(2):1044. doi: 10.3390/ijms24021044 (PMC9862653; doi:10.3390/ijms24021044)
Supplement: Supplementary file 1 [file ijms-24-01044-s001.zip › ijms-2067192-supplementary.pdf]

SUPPLEMENTARY MATERIAL

Exploring the effects of Metabolism-Disrupting Chemicals on Pancreatic α-Cell Viability, Gene Expression and Function: A Screening Testing Approach

Ruba Al-Abdulla[1#], Hilda Ferrero[1,2#], Talía Boronat-Belda[1], Sergi Soriano[1,3]

Iván Quesada[1,2] and Paloma Alonso-Magdalena[1,2*]

[1] Instituto de Investigación, Desarrollo e Innovación en Biotecnología Sanitaria de Elche (IDiBE), Universidad Miguel Hernández, Elche, Spain.

[2] Centro de Investigación Biomédica en Red de Diabetes y Enfermedades Metabólicas Asociadas (CIBERDEM), Spain.

[3] Departamento de Fisiología, Genética y Microbiología, Universidad de Alicante, Alicante, Spain.

*Author to whom correspondence should be addressed: Paloma Alonso-Magdalena. palonso@umh.es

# These authors contributed equally to this work

48 h	RZ	NRU	CFDA-AM
Control	100.00 ± 1.72	100.00 ± 0.94	100.00 ± 1.01
BPA 100 pM	94.13 ± 2.04	98.74 ± 1.23	96.92 ± 0.84
BPA 1 nM	92.42 ± 2.00 *	96.31 ± 0.96	96.44 ± 1.02
BPA 10 nM	93.27 ± 2.18	97.58 ± 0.87	96.97 ± 1.35
BPA 100 nM	90.86 ± 2.00 **	97.12 ± 1.03	94.27 ± 0.87 **
BPA 1 µM	91.56 ± 1.76 *	95.43 ± 1.19 *	94.54 ± 1.24 **
BPA 10 µM	96.26 ± 1.90	93.80 ± 1.49 ***	93.70 ± 1.18 ***
	RZ	NRU	CFDA-AM
Control	100.00 ± 1.15	100.00 ± 1.49	100.00 ± 0.69
BPS 100 pM	100.85 ± 1.61	103.46 ± 1.36	98.92 ± 0.61
BPS 1 nM	97.83 ± 1.14	103.67 ± 0.96	99.32 ± 0.76
BPS 10 nM	100.93 ± 1.36	102.61 ± 1.32	100.57 ± 0.49
BPS 100 nM	100.22 ± 1.91	103.85 ± 1.67	99.27 ± 0.94
BPS 1 µM	97.91 ± 2.07	103.17 ± 1.12	98.23 ± 1.02
BPS 10 µM	89.91 ± 3.72	90.34 ± 4.04	97.53 ± 2.04
	RZ	NRU	CFDA-AM
Control	100.00 ± 0.61	100.00 ± 0.94	100.00 ± 0.84
BPF 100 pM	96.66 ± 1.03	98.54 ± 1.11	97.70 ± 0.95
BPF 1 nM	93.63 ± 1.18 ***	97.57 ± 1.30	94.51 ± 0.97 **
BPF 10 nM	91.94 ± 1.09 ****	96.39 ± 1.02	93.84 ± 1.00 ****
BPF 100 nM	92.35 ± 1.02 ****	98.62 ± 1.01	92.49 ± 0.82 ****
BPF 1 µM	90.57 ± 1.15 ****	97.90 ± 1.18	91.45 ± 1.06 ****
BPF 10 µM	86.61 ± 1.27 ****	95.01 ± 1.03 *	91.27 ± 1.15 ****
	RZ	NRU	CFDA-AM
Control	100.00 ± 0.87	100.00 ± 0.75	100.00 ± 0.91
DEHP 100 pM	96.88 ± 0.84	99.16 ± 0.46	98.62 ± 0.79
DEHP 1 nM	93.80 ± 1.27 **	99.69 ± 0.88	96.72 ± 0.97
DEHP 10 nM	94.70 ± 1.30 *	99.53 ± 1.40	96.16 ± 0.97 *
DEHP 100 nM	93.77 ± 1.30 **	99.08 ± 0.68	96.01 ± 1.02 *
DEHP 1 µM	93.25 ± 1.42 **	100.09 ± 1.19	94.73 ± 0.94 ***
DEHP 10 µM	106.54 ± 1.83 **	97.31 ± 0.76	95.02 ± 0.94 **
	RZ	NRU	CFDA-AM
Control	100.00 ± 0.85	100.00 ± 1.33	100.00 ± 4.02
PFOS 100 pM	96.39 ± 1.33	98.99 ± 1.14	97.94 ± 1.15
PFOS 1 nM	91.99 ± 1.84 **	97.62 ± 1.01	96.36 ± 1.73
PFOS 10 nM	85.62 ± 2.37 ****	88.47 ± 3.45 ****	93.34 ± 1.92 *
PFOS 100 nM	91.97 ± 1.56 **	97.37 ± 0.63	93.56 ± 2.08 **
PFOS 1 µM	89.13 ± 1.34 ****	97.42 ± 1.49	93.26 ± 0.61 **
PFOS 10 µM	93.94 ± 1.63 *	97.20 ± 1.84	91.74 ± 1.07 ***
	RZ	NRU	CFDA-AM
Control	100.00 ± 1.29	100.00 ± 0.92	100.00 ± 0.88
$CdCl_2$ 100 pM	98.90 ± 1.35	99.56 ± 1.25	98.67 ± 0.99
$CdCl_2$ 1 nM	98.30± 1.56	98.12 ± 1.43	98.12 ± 1.19
$CdCl_2$ 10 nM	97.55 ± 1.58	97.85 ± 1.22	97.17 ± 1.20
$CdCl_2$ 100 nM	97.82 ± 1.67	95.41 ± 1.29 *	98.02 ± 1.19
$CdCl_2$ 1 µM	96.31 ± 1.69	92.60 ± 1.73 ***	96.39 ± 1.37
$CdCl_2$ 10 µM	95.16 ± 1.76	94.86 ± 1.09 *	95.93 ± 1.39
	RZ	NRU	CFDA-AM
Control	100.00 ± 0.69	100.00 ± 0.52	100.00 ± 0.50
DDE 100 pM	96.04 ± 1.16 *	98.81 ± 1.00	97.99 ± 0.94
DDE 1 nM	94.26 ± 1.16 ***	98.33 ± 0.68	96.06 ± 0.44
DDE 10 nM	94.00 ± 1.32 ***	99.19 ± 0.81	94.19 ± 1.14 ***
DDE 100 nM	94.96 ± 1.04 **	101.68 ± 0.70	95.44 ± 1.22 *
DDE 1 µM	95.03 ± 1.24 **	101.69 ± 0.86	94.13 ± 1.20 ***
DDE 10 µM	95.87 ± 1.04 *	103.55 ± 0.74 **	90.60 ± 1.19 ****

Supplemental Table S1. Viability of pancreatic αTC1-9 cells treated for 48 h with different BPA, BPS, BPF, DEHP, PFOS, $CdCl_2$, or DDE concentrations (100 pM–10 µM) as evaluated by RZ, NR and CFDA-AM assays. n= at least 3 independent experiments. All data are expressed as mean ± SEM. *vs. Control; *p < 0.05, **p < 0.01, ***p < 0.001 and ****p < 0.0001 by one-way ANOVA followed by Dunnet's post hoc test or Kruskal-Wallis followed by Dunn's post hoc test.

72 h	RZ	NRU	CFDA-AM
**Control**	100.00 ± 1.28	100.00 ± 0.64	100.00 ± 0.75
**BPA 100 pM**	98.51 ± 1.53	100.09 ± 1.28	100.00 ± 0.99
**BPA 1 nM**	94.97 ± 1.43	100.27 ± 1.38	98.49 ± 1.11
**BPA 10 nM**	96.22 ± 1.79	98.61 ± 1.34	97.86 ± 0.92
**BPA 100 nM**	97.53 ± 1.61	100.68 ± 1.30	98.22 ± 0.75
**BPA 1 µM**	95.76 ± 0.94	101.80 ± 1.26	99.59 ± 0.94
**BPA 10 µM**	93.17 ± 1.04 **	97.64 ± 1.45	97.36 ± 1.43
	**RZ**	**NRU**	**CFDA-AM**
**Control**	100.00 ± 1.07	100.00 ± 0.70	100.00 ± 0.83
**BPS 100 pM**	98.49 ± 1.03	97.28 ± 0.88	99.50 ± 0.80
**BPS 1 nM**	96.04 ± 1.11 *	94.84 ± 1.21 **	98.46 ± 0.58
**BPS 10 nM**	95.03 ± 1.61 *	98.90 ± 1.52	98.73 ± 1.07
**BPS 100 nM**	97.62 ± 0.95	99.84 ± 1.70	97.76 ± 0.94
**BPS 1 µM**	97.87 ± 1.84	98.54 ± 1.47	99.34 ± 1.26
**BPS 10 µM**	89.82 ± 2.97 *	96.58 ± 2.50	97.28 ± 0.89
	**RZ**	**NRU**	**CFDA-AM**
**Control**	100.00 ± 0.61	100.00 ± 0.52	100.00 ± 0.54
**BPF 100 pM**	95.10 ± 0.61 *	98.55 ± 1.05	97.90 ± 0.71
**BPF 1 nM**	95.16 ± 1.04 *	98.99 ± 0.70	97.29 ± 0.63 *
**BPF 10 nM**	92.76 ± 1.04 ****	97.12 ± 0.99	96.21 ± 1.04 **
**BPF 100 nM**	93.14 ± 1.23 ****	97.44 ± 1.04	95.95 ± 0.88 **
**BPF 1 µM**	92.30 ± 1.30 ****	95.83 ± 1.05	95.47 ± 0.85 ***
**BPF 10 µM**	90.83 ± 1.43 ****	95.24 ± 1.16	95.40 ± 0.84 ***
	**RZ**	**NRU**	**CFDA-AM**
**Control**	100.00 ± 1.39	100.00 ± 0.87	100.00 ± 1.16
**DEHP 100 pM**	95.94 ± 1.67	99.99 ± 1.20	95.93 ± 1.36
**DEHP 1 nM**	93.96 ± 1.49 *	99.03 ± 1.11	94.08 ± 1.34 *
**DEHP 10 nM**	91.38 ± 1.07 ***	99.33 ± 1.37	94.65 ± 1.33 *
**DEHP 100 nM**	93.84 ± 1.61 *	99.07 ± 0.97	94.07 ± 1.52 *
**DEHP 1 µM**	94.53 ± 1.45 *	101.80 ± 1.23	92.49 ± 1.39 ***
**DEHP 10 µM**	105.00 ± 1.66	97.78 ± 1.14	92.78 ± 1.30 **
	**RZ**	**NRU**	**CFDA-AM**
**Control**	100.00 ± 1.75	100.00 ± 1.97	100.00 ± 1.10
**PFOS 100 pM**	98.71 ± 1.15	95.05 ± 2.08	97.57 ± 0.92
**PFOS 1 nM**	98.32 ± 0.81	94.99 ± 2.55	97.80 ± 0.64
**PFOS 10 nM**	82.48 ± 3.25 ****	82.20 ± 3.66 ***	90.85 ± 1.83 ****
**PFOS 100 nM**	93.06 ± 2.72	92.35 ± 2.94	95.84 ± 1.38 *
**PFOS 1 µM**	95.63 ± 1.19	88.27 ± 2.60 **	96.38 ± 0.83 *
**PFOS 10 µM**	99.78 ± 2.33	91.78 ± 2.09 *	98.87 ± 1.01
	**RZ**	**NRU**	**CFDA-AM**
**Control**	100.00 ± 0.96	100.00 ± 2.12	100.00 ± 0.68
**$CdCl_2$ 100 pM**	100.90 ± 1.20	101.00 ± 2.30	99.63 ± 0.76
**$CdCl_2$ 1 nM**	99.99 ± 0.97	100.90 ± 2.61	99.07 ± 0.90
**$CdCl_2$ 10 nM**	97.89 ± 1.26	102.90 ± 2.61	98.25 ± 0.86
**$CdCl_2$ 100 nM**	100.60 ± 1.00	102.10 ± 2.35	98.62 ± 1.29
**$CdCl_2$ 1 µM**	98.95 ± 1.04	101.20 ± 1.53	97.93 ± 1.16
**$CdCl_2$ 10 µM**	95.61 ± 1.45 *	99.33 ± 1.56	97.10 ± 1.27
	**RZ**	**NRU**	**CFDA-AM**
**Control**	100.00 ± 0.46	100.00 ± 0.58	100.00 ± 0.50
**DDE 100 pM**	97.84 ± 0.43 **	97.93 ± 0.57	98.21 ± 0.59
**DDE 1 nM**	98.75 ± 0.54	99.33 ± 0.62	98.91 ± 0.53
**DDE 10 nM**	98.30 ± 0.56	99.43 ± 0.69	97.87 ± 0.66
**DDE 100 nM**	98.10 ± 0.61 **	99.74 ± 0.65	97.33 ± 0.59 **
**DDE 1 µM**	97.26 ± 0.44 ***	99.90 ± 0.61	97.21 ± 0.57 **
**DDE 10 µM**	97.02 ± 0.49 ***	100.10 ± 0.89	96.28 ± 0.59 ****

Supplemental Table S2. Viability of pancreatic αTC1-9 cells treated for 72 h with different BPA, BPS, BPF, DEHP, PFOS, $CdCl_2$, or DDE concentrations (100 pM–10 µM) as evaluated by RZ, NR and CFDA-AM assays. n= at least 3 independent experiments. All data are expressed as mean ± SEM. *vs. Control; *$p < 0.05$, **$p < 0.01$, ***$p < 0.001$ and ****$p < 0.0001$ by one-way ANOVA followed by Dunnet's post hoc test, or Kruskal-Wallis followed by Dunn's post hoc test.

Gene	Forward (5´→ 3´)	Reverse (5´→ 3´)
Gcg	CACTCACAGGGCACATTCAC	TTTGGCAATGTTGTTCCGGTT
Gck	TTCAGCTTCTGGCCTCCCACAG	AAAACAGCCAGGTCTGGGCAGC
Glut1	GTGTCGCTGTTTGTTGTAGAG	CAAAGCCAAAGATGGCCACGA
Arx	GGCCGGAGTGCAAGAGTAAAT	TGCATGGCTTTTTCCTGGTCA
MafB	ACCAAGGACGAGGTGATCC	CAGGTGATGTTTCTGCTGGA
Foxo1	AAGAGCGTGCCCTACTTCAA	CTCTTGCCCAGACTGGAGAG
Hprt	GGTTAAGCAGTACAGCCCCA	TCCAACACTTCGAGAGGTCC
Actb	GGCTGTATTCCCCTCCATCG	CCAGTTGGTAACAATGCCATGT
Gapdh	ACACTGAGCAAGAGAGGCCCTA	GGGTGCAGCGAACTTTATTGATGGTATT

Supplemental Table S3. Primer sequences used in RT-qPCR for the study of pancreatic α-cell gene expression.
